# Aesthetic reconstruction of onco-surgical maxillary defects using free scapular flap with and without CAD/CAM customized osteotomy guide

**DOI:** 10.1186/s12893-022-01811-9

**Published:** 2022-10-19

**Authors:** Mohamed Salah Alwadeai, Leena Ali Al-aroomy, Mostafa Ibrahim Shindy, Ayman Abdel-Wahab Amin, Mohamed Hamdallah Zedan

**Affiliations:** 1 Department of Oral and Maxillofacial Surgery, Faculty of Dentistry, Cairo University, Elmanial Street, Cairo, Egypt; 2 Department of Oral and Maxillofacial Pathology, Faculty of Dentistry, Cairo University, Cairo, Egypt; 3 Department of Surgical Oncology-National Cancer Institute, Cairo University, Cairo, Egypt; 4 Faculty of Dentistry, Ibb University, Ibb, Yemen

**Keywords:** Scapula, Surgical guide, Flap, Maxillary defect, Computer-aided design, Computer-aided manufacturing, Operation time

## Abstract

**Background:**

Reconstruction of the maxillary defects following tumor ablation remains to be a challenge for surgeons. Virtual surgical planning (VSP), intraoperative cutting guides and stereolithographic models provides the head and neck reconstructive surgeon with powerful tools for complex reconstruction planning. Despite its use in fibular osteocutaneous reconstruction, application to the scapular free flap has not been as widely reported. The aim of this study was to compare aesthetic results and operation time of free scapular flap, with and without computer-aided design and computer-aided manufacturing (CAD/CAM) customized osteotomy guide, for reconstruction of maxillary defects.

**Methods:**

In this prospective randomized controlled clinical trial study, we randomly assigned 22 patients with maxillary defects who were scheduled to undergo maxillary reconstruction with either free scapular flap with CAD/CAM customized osteotomy guide; study group (n = 11) or free scapular flap without CAD/CAM customized osteotomy guide; control group (n = 11). All patients were followed up for 3 months. The aesthetic outcome, operation and ischemic times were evaluated and compared.

**Results:**

Aesthetic outcomes were evaluated objectively by means of differential surface area (sagittal and axial) measurement, which showed improved aesthetic outcome (contour symmetry) in the study group with a mean of (241.39 ± 113.74 mm^2^), compared to patients in control group with a mean of (358.70 ± 143.99 mm^2^). There were significant differences between the two groups (P < 0.05). Aesthetic outcomes were also evaluated subjectively by means of visual analogue scale (VAS) and patient’s satisfaction score (PSS). The postoperative aesthetic appearance was better in the study group with more patients satisfied than that in control group and there were statistically significant differences (P = 0.039). The mean total operative time was 678.81 min and 777.18 min in the study group and control group respectively (P < 0.05) and the mean ischemia time was 133.18 min and 195.72 min for study group and control group respectively (P < 0.05). The ischemia time and total operative time were shorter in the study group compared to those in the control group and there were no flap losses in both groups.

**Conclusion:**

The use of CAD/CAM prefabricated cutting guides facilitates scapular flap molding and placement, minimizes ischemic time and operating time as well as improves aesthetic outcomes.

*Trial* *registration* This trial was registered at ClinicalTrials.gov. Registration number: NCT03757286. Registration date: 28/11/2018

## Background

Maxillary defects represent a unique challenge for reconstructive surgeons. Resection of neoplasms in the maxilla often results in complex defects encompassing soft tissue, bone and dentition. This results in diminished aesthetics and impaired oral functions and thereby lowers the quality of life [1].

Correction of the maxillary defect can be done either by a surgical procedure or by prosthetic rehabilitation. Various free tissue transfers have been advocated for palatal and midfacial reconstruction, including scapular, fibular, radial forearm, rectus abdominus, iliac crest, and latissimus dorsi flaps [2].

Scapular flaps have been discussed vastly in the literature with several different applications. Scapular bone has been reported to provide sufficient bone and soft tissue components to reconstruct maxillectomy defects. Some authors considered it to be the first reconstructive option in cases of maxillectomy with orbital exenteration defects. The scapula has been used for maxillary reconstruction and offers several advantages when compared to other reconstructive options. In reconstructing a hemimaxillectomy defect, the advantage of the scapular tip free tissue transfer is the shape of the osseous component of the flap and how it mirrors the shape of the resected maxilla and palate [3, 4].

Conventional reconstructive methods often lead to inaccurate reconstructions due to inexact planning, failed communication between the resective and reconstructive teams or surgical difficulties in adjusting a free flap and osteosynthesis plates into a three-dimensional (3D) defect without the help of any templates or surgical guides [5].

Recent advances in computer-aided design and manufacturing software have allowed surgeons to achieve this goal and perform tumor resection and maxillofacial reconstruction with greater accuracy. Virtual surgical planning (VSP) software has allowed surgeons to perform virtual surgery preoperatively and generate templates and cutting guides to improve intraoperative precision. As a result, surgeons can perform accurate tumor resection, flap osteotomy and maxillary reconstruction and reduce operative time [6, 7]. The purpose of this study was to compare aesthetic results and operation time of free scapular flap with and without CAD/CAM customized osteotomy guide for reconstruction of maxillary defects.

## Methods

### Trial design and participants

This prospective randomized controlled clinical trial study was designed according to the CONSORT checklist and was done between December 2018 and February 2021 in Department of Head and Neck, National Cancer Institute, Cairo University, Egypt. Twenty-two patients were randomized in equal proportions between two groups; a study group: maxillary reconstruction using free scapular flap with CAD/CAM customized osteotomy guide and a control group: maxillary reconstruction using free scapular flap without customized osteotomy guide (model-based reconstruction). Both surgical procedures were performed by the same chief surgeon.

Inclusion criteria included: Patients affected by tumor involving maxillary bone.

Exclusion criteria included: Patients having poor oncological prognosis and patients with poor performance status together with other relative or absolute vascular contraindication.

### Operative procedures

#### Virtual surgical planning

VSP consisted of a surgical planning session through which the whole process was planned virtually. This was followed by the design phase CAD, ending with the production phase CAM in which custom-made osteotomy guides and models were fabricated. The process of virtual planning began with acquisition of high-resolution computed tomography (CT) scans for craniofacial skeleton and scapula. Images were saved in Digital Imaging and Communications in Medicine (DICOM) format. The CT scanned images in DICOM format were processed using synthes ProPlan CMFTM_software (Materialise, Technologielaan 15, 3001 Leuven, Belgium http://www.materialise.com).

**Fig. 1:**
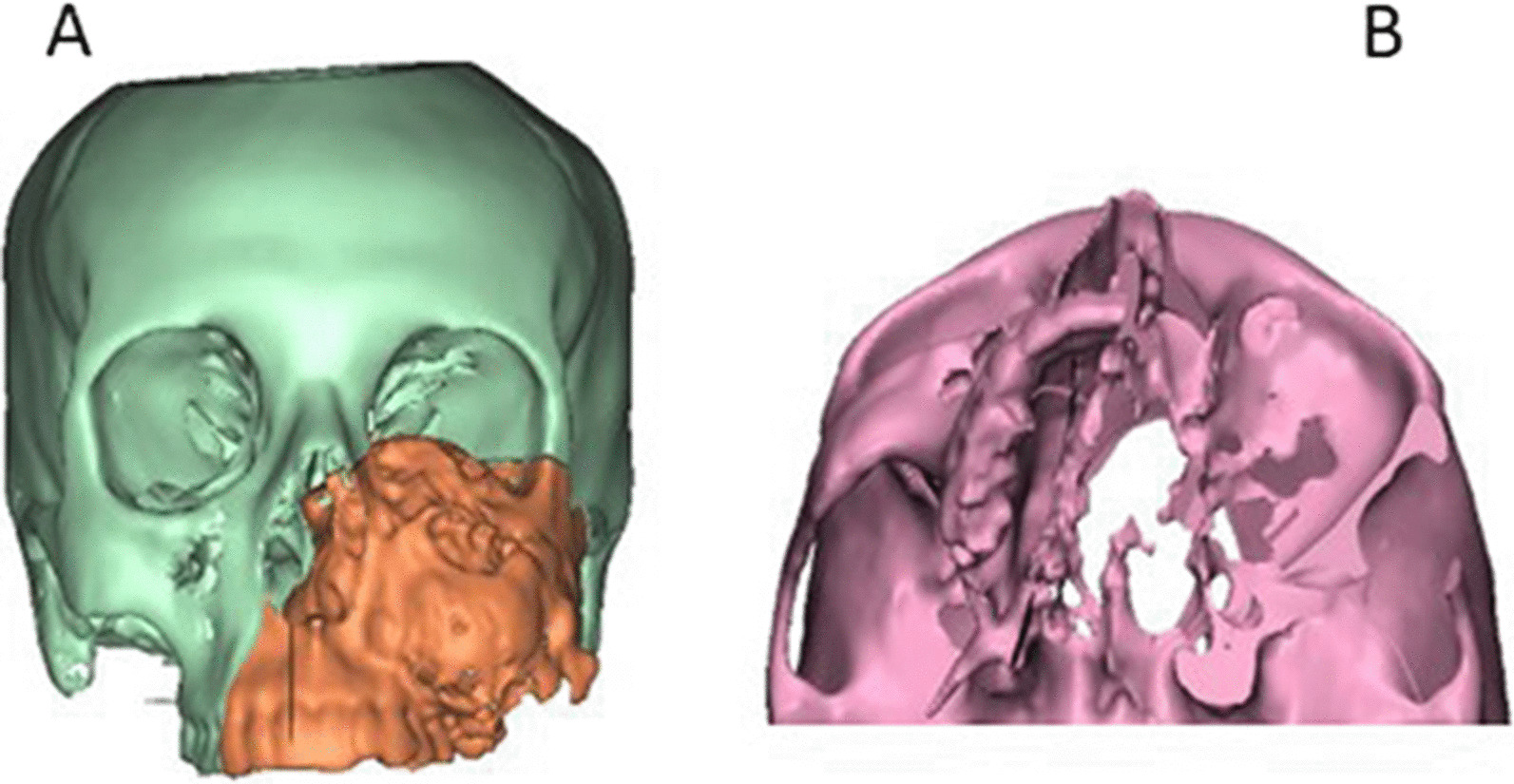
**A**, **B** Showing 3D virtual models of the maxillofacial skeleton with expected bone defect

Firstly, segmentation process for the region of interest was accomplished and transformed for creation of 3-dimensional (3D) virtual models of the maxillofacial skeleton and the bony scapula, which represented the basis for the virtual planning of the surgical procedures (Fig. 1).

#### Virtual resection phase for the maxilla

**Fig. 2:**
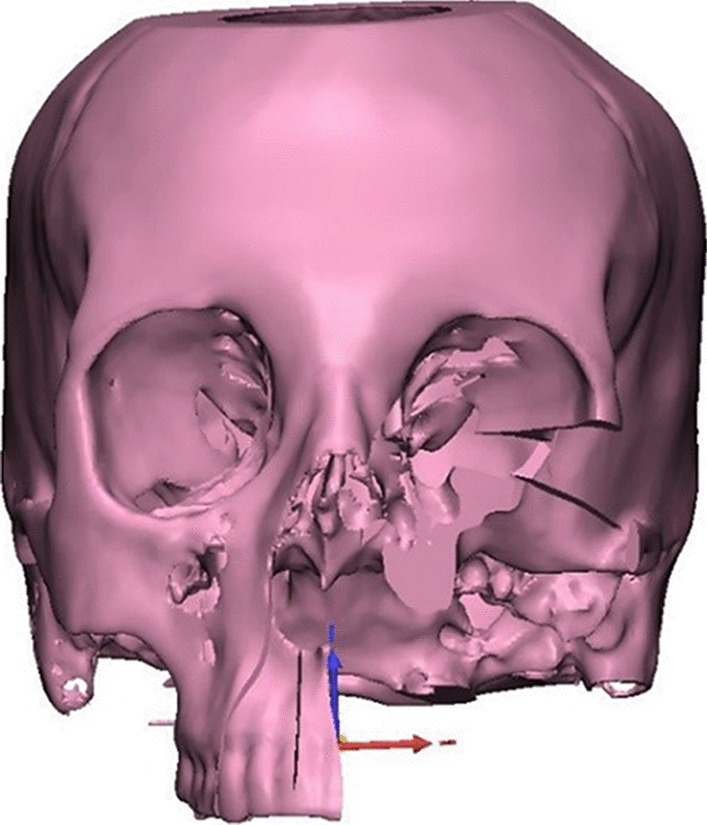
Virtual planned of maxillary defect resection

Virtual maxillectomy was performed with ProPlan CMFTM (Materalise, Leuven, Belgium) based on the predetermined plan (Fig. 2) and the mapped osteotomies that had been planned according to the principles of radical tumor resection, which were confirmed in the communication session.

#### Virtual reconstruction phase using scapula

For the study group, the 3-dimensional virtual models were reviewed by the reconstructive surgeon and used to plan the size and shape of scapular bone cuts.

**Fig. 3:**
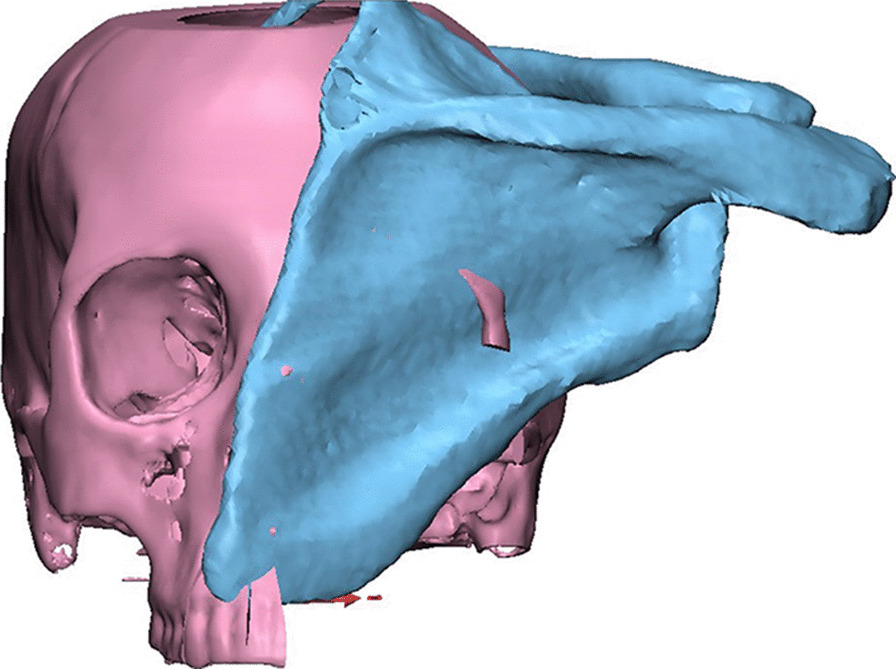
3D reconstructed scapula was superimposed on the maxillary defect

**Fig. 4:**
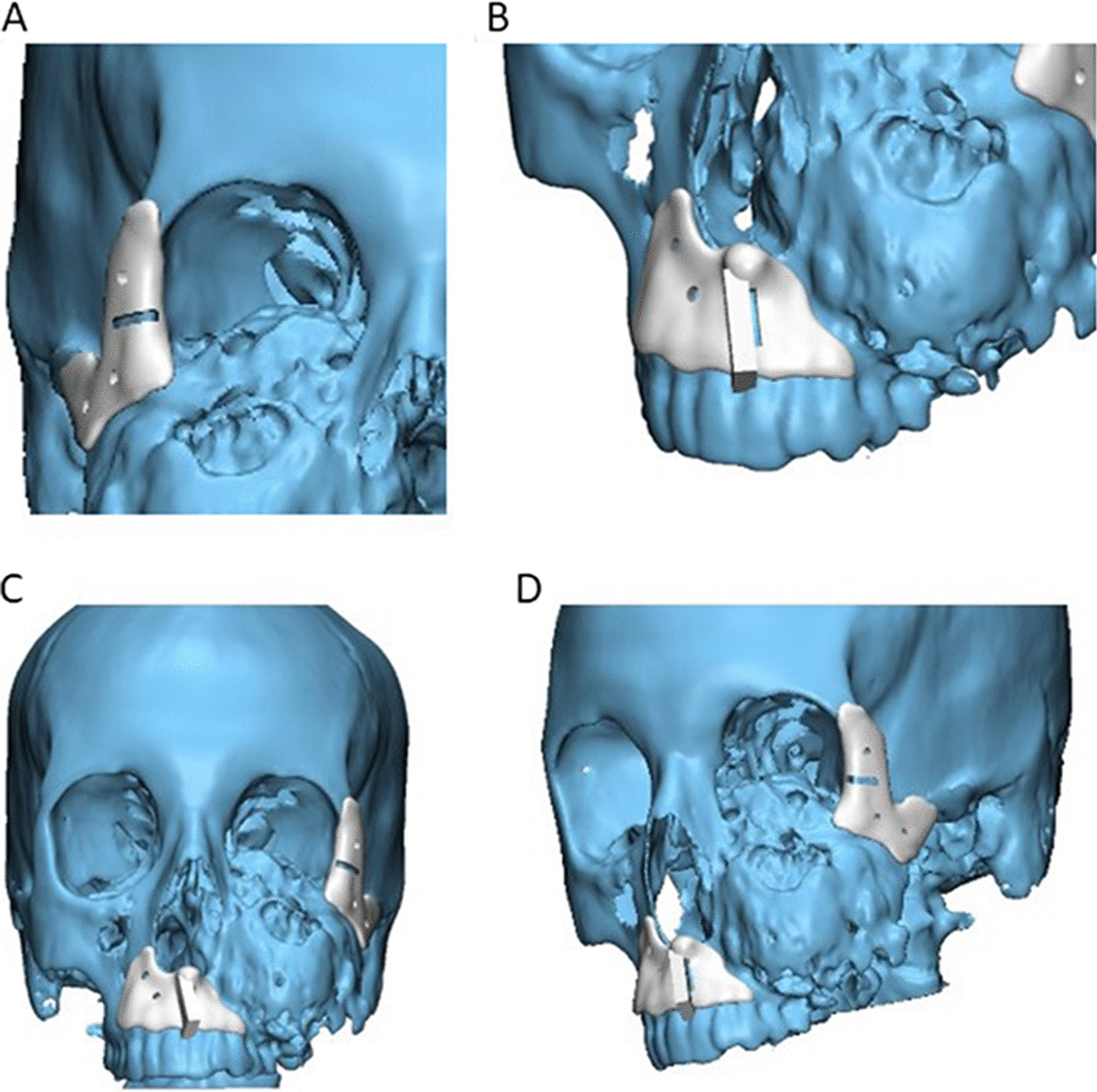
**A**–**D** Different views of 3D virtually designed maxillary resection (cutting) guide

**Fig. 5:**
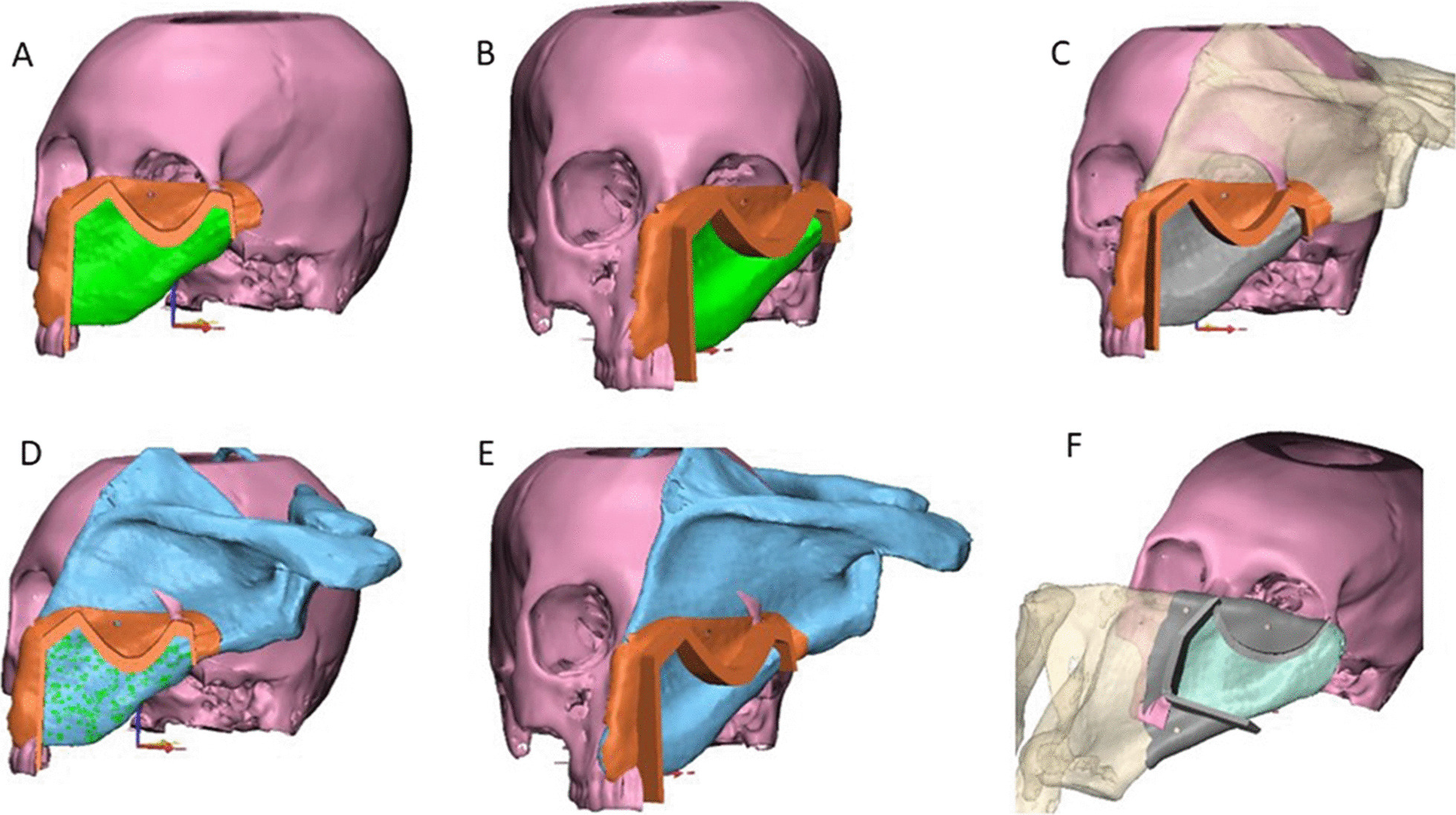
**A**–**F** Different views of 3D virtually designed scapular (osteotomy) cutting guide

Next, the patient’s 3D reconstructed scapula was superimposed on the maxillary defect, and scapular osteotomies were placed to recreate the native maxillary contour through a trial-and-error process for shaping and placement of scapular bone which was made (Fig. 3). The scapula segment lengths, the number of osteotomies and bone-to-bone contact were evaluated. After that, 3D-model for the virtually reconstructed maxilla with scapula was created. Once the virtual planning was completed, the virtual resection/reconstruction data were used to design autoclavable custom-made surgical cutting guides for the planned resection and scapular osteotomies (Figs. 4 and 5).

**Fig. 6:**
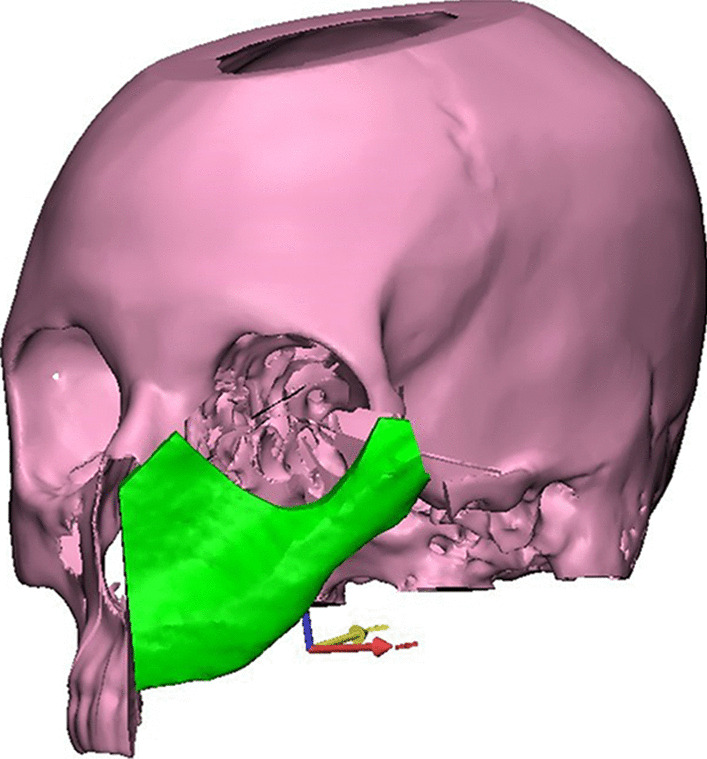
Proposed virtually scapular reconstruction of maxillary defect

Following this, the surgical guides and the reconstructed maxillary models were saved in stereolithography (STL) format and then imported into a rapid prototyping machine (Wiiboox; JOC, Jiangsu, China) for 3D printing into real 1:1 model (Fig. 6). Finally, the printed surgical cutting guides for both maxilla and scapula, in addition to the 3D reconstructed maxillary models, were sent to be sterilized.

### Surgical phase

#### In the study group

##### Ablative surgery of maxilla

After the surgical fields were exposed and the tumor was approached and explored, the customized 3D maxillary cutting guides were then applied and secured with screws in the pre-designated planned position to determine the resection margins without interfering with the tumor. This ensured precise guiding for the surgeon throughout the process of maxillary resection. The osteotomies were performed using Lindemann bur inserted through the inherent cutting slots to resect the tumor, replicating the virtually planned maxillary resection.

##### Free scapular flap harvesting

To harvest the scapular flap, the patient was brought in a lateral decubitus position. The skin incision and the flap design depended on the type of flap that was harvested. The osteotomies of scapula were performed according to the preoperative planning, using the CAD/CAM fabricated cutting guides. Before cutting the scapula, the cutting guide was temporarily placed on the scapula to assess and confirm the accurate position. After confirming that the cutting guide was fixed accurately and fitted to the scapula, the scapula was cut along the guide using Lindemann bur, which was inserted into slots, and deperiostation was performed. The flap was then completely mobilized and the pedicle was transected when the recipient site was ready. To incorporate the harvested flap and complete the reconstruction, the scapula and plate were fixed to the native maxilla segments at their virtually planned ideal position, ensuring extremely precise bone to bone contact using miniplate and screw.

#### In the control group

The same procedure for resection and reconstruction was performed, except that no maxillary and scapular customized osteotomy guide was used. Instead, only 3D model for maxilla was obtained by virtual planning and mirroring the intact side to aid in plate shaping and positioning. The shaped scapular flap was then secured to the pre-bent plate conformed on the contours of the 3D model.

### Outcome evaluation phase

#### Aesthetic evaluation (primary outcomes)

##### Objective evaluation

The post-operative surgical outcomes (aesthetic) were evaluated objectively by acquisition of high-resolution CT scan to be used in CDIA. The measurements were then calculated and compared as differential area in square millimeter (mm^2^).

### Differential area measurement

**Fig. 7:**
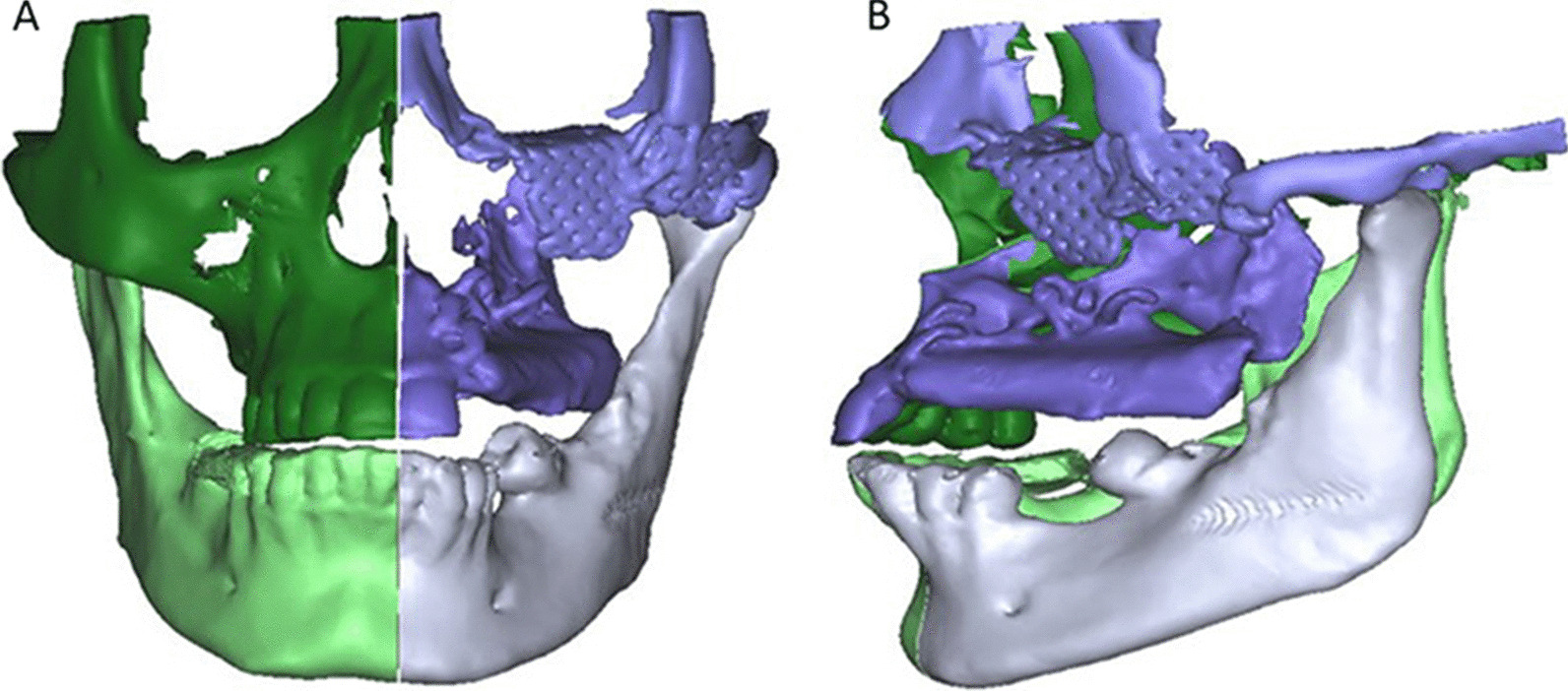
**A** Frontal and **B** lateral view of 3D virtual models of the maxillofacial skeleton

The CT scanned images in DICOM format were processed using ProPlan CMF_ software (Materialise, Technologielaan 15, 3001 Leuven, Belgium http://www.materialise.com) for production of 3-dimensional (3D) virtual models of the maxillofacial skeleton (Fig. 7).

**Fig. 8:**
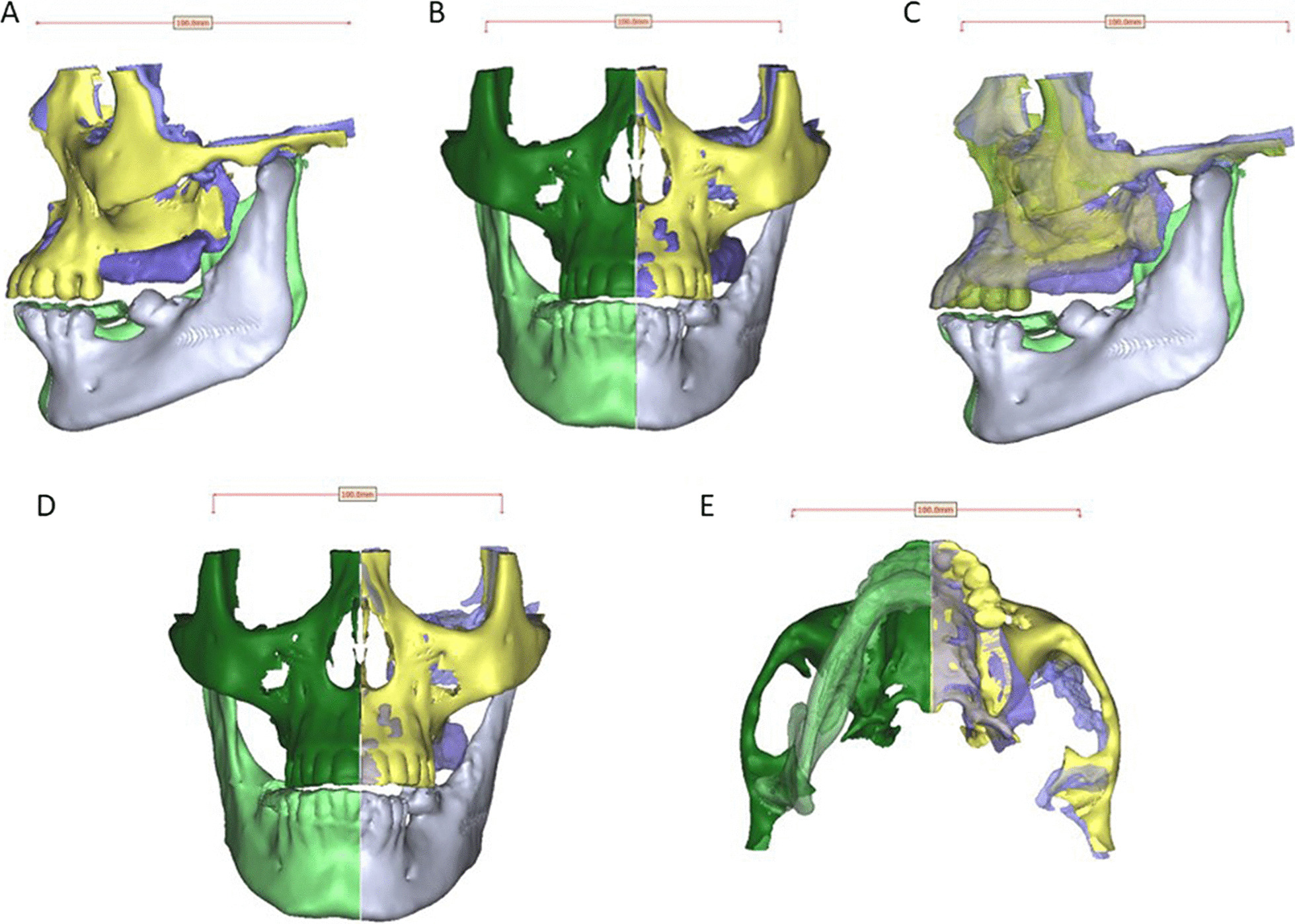
**A**-**E** Transparency function applied to the Superimposition between the affected and contralaetral sides in different views

The contralateral side was simply mirrored and superimposed on affected side of the obtained 3D model of the maxilla. After performing superimposition between the reconstructed side and the contralateral side, a digital reference was placed to be used in calibration during measurements in square millimeters. The resultant superimposition in sagittal and axial views, including the digital reference, was saved as 2D image to be used for surface area measurement using ImageJ program (Fig. 8).

**Fig. 9:**
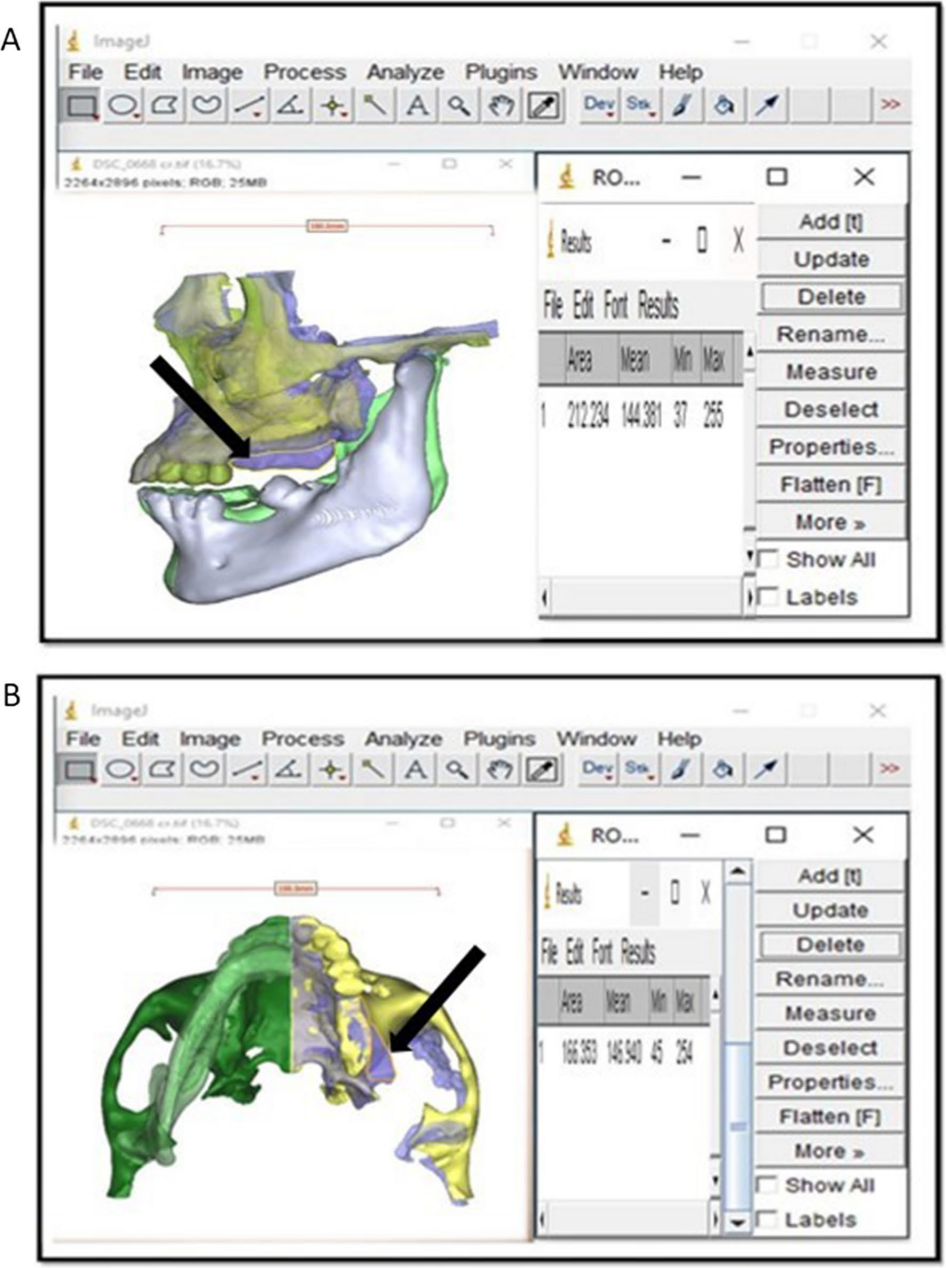
Screenshot showing the ImageJ computer program, designed for measurement of differential surface area (black arrow) in **A** sagittal and **B** axial aspects

The obtained 2D image was imported to the ImageJ software and the differential surface area was then traced and determined using polygon selections tool in the ImageJ. The differential area was measured and calculated to evaluate aesthetic outcomes, using the measure function tool in the ImageJ computerized digital program, after performing calibration using the digital reference placed previously (Fig. 9).

### Subjective evaluation using VAS

The patients were assessed by two evaluators; the surgeon and the maxillofacial professional. They evaluated the aesthetic outcome seen clinically on patients in different sequences and indicated their grade using a visual analogue scale sheet, which ranged from 0 to 10; with zero representing the poorest aesthetic outcome and 10 representing the best outcome. The values were then compared between the two groups.

### Subjective evaluation using PSS

Subjective evaluation was also carried out using patients’ perceptions of the aesthetic outcome, measured by the degree in which patients expressed their satisfaction or dissatisfaction. The results were expressed in numbers from zero (worst aesthetic) to 10 (best aesthetic). The values were then compared between the two groups.

### Operation time evaluation (secondary outcomes)

Operation time was assessed as total operation time and ischemia time. Total operative time was the duration from surgical incision to wound closure, which included the time for tumor excision, neck dissection, flap elevation, and reconstructions. On the other hand, ischemic time was the duration from the time the flap’s pedicle was divided to the time when anastomosis was performed. Operation time was assessed in the surgery day, intraoperatively, using a digital watch. The entire procedure was timed and recorded on a timing sheet and the times of the entire procedure were measured in minutes as a final value and summarized as a mean value.

#### Primary outcome

The primary objective of the study was to compare aesthetic results of free scapular flap with and without CAD/CAM customized osteotomy guide for reconstruction of maxillary defects. The post-operative surgical aesthetic outcomes were evaluated objectively by measuring of differential surface area of the maxilla using Computerized Digital Imaging Analysis (CDIA) and subjectively using Visual Analogue Scale (VAS) and Patient’s Satisfaction Score (PSS). This evaluation was performed at three months postoperatively.

#### Secondary outcome

The secondary objective of the study was to compare operation time of free scapular flap with and without CAD/CAM customized osteotomy guide for reconstruction of maxillary defects. Operation time was assessed as total operation time and ischemia time.

#### Sample size

Based on a previous study by Azuma et al., 2014, the clinical significant difference in differential angle between two groups was expected to be 6̊, with standard deviation of control group was 4.38̊. Using power 80% and 5% significance level, we thus needed to study nine patients in each group (Total: 18). This number was increased to a sample size of 11 in each group (Total: 22) to compensate for losses during follow up (20% more than the calculated). Sample size was calculated using PS program.

### Randomization

#### Methods of generating allocation sequence

The total sample size (n = 22) and the sample sizes in each group were pre-specified exactly and were under the direct control of the investigator. In this case, the usual simple randomization procedure was the random allocation rule, and allocation ratio (1:1) was used. Accordingly, a randomly chosen subset of (n/2 = 11) out of (n = 22) was assigned to group (a) (study group), taking a sequential numbering (1a, 2a, 3a, 4a, 5a, 6a, 7a, 8a, 9a, 10a, 11a), whereas the remainder were assigned to group (b) (control group) with sequential numbering (12b, 13b, 14b, 15b, 16b, 17b, 18b, 19b, 20b, 21b, 22b).

#### Allocation concealment mechanism

Twenty two cards took the generated sequence numbers, with one number for each card, then these cards were placed within opaque sealed envelopes, which were subsequently placed in a container (box). Each participant grasped one envelope blindly on the day of the operation.

#### Implementation

Principal investigator (MA) was the person who generated the allocation sequence, enrolled the participants and assigned the participants for intervention.

#### Masking/blinding

Because the two interventions used in this trial were easily recognized by the participants and the investigator, neither investigator (MA) nor participant could be blinded. Besides, blinding the assessor (MS) was not possible, unlike the statistician who was successfully blinded.

### Statistical analysis

IBM SPSS advanced statistics (Statistical Package for Social Sciences), version 24, was used to examine the data (SPSS Inc., Chicago, IL). The mean, standard deviation, or median and range were used to characterise numerical data. Numbers and percentages were used to define categorical data. Shapiro–Wilk and Kolmogrov–Smirnov tests were used to examine the data for normality. For comparisons between two groups involving regularly distributed numerical variables, the Student’s t-test was used, whereas the Mann–Whitney test was used for comparisons involving non-normally distributed numerical variables. The Chi-square test was used to make comparisons between category variables. Statistical significance was defined as a p-value ≤ 0.05.

## Results

### Patients’ demographic data

A total of 22 patients with maxillary tumors were randomly divided into two equal groups; where the study group (11 patients) was treated with the CAD/CAM osteotomy cutting guide approach and the control group (11 patients) was treated with the CAD/CAM without osteotomy cutting guide. In the study group, the mean age was (48.36 ± 14.14) years (range, 28–71 years), while in the control group, the mean age was (50.09 ± 17.14) years (range, 25–71 years). There were four males (36.36%) and seven females (63.63%) in the study group and there were five males (45.45%) and six females (54.54%) in the control group.

Of 22 patients, 10 patients had squamous cell carcinoma, four patients had osteosarcoma, three patients had adenoid cystic carcinoma, three patients had ameloblastoma, one patient had mucoepidermoid carcinoma and one had central ossifying fibroma. The mean defect lengths in the study and control groups were (7.92 ± 1.49 mm) and (8.80 ± 1.34 mm) respectively with the P value of [0.161]. None of these patients had previously been surgically treated for tumors nor had they undergone any major surgery previously. All patients had completed the follow up period (3 months). The clinicodemographic characteristics of both groups are presented in Table 1.

**Table 1:** Clinicodemographic characteristics of both groups of patients enrolled in this study

	Study group		Control group		
	Count	%	Count	%	
Sex					
Male	4	36.36	5	45.45	
Female	7	63.63	6	54.54	
Age	Mean ± SD		Mean ± SD		P value
	48.36 ± 14.14		50.09 ± 17.14		0.799
Pathology					
Benign	2	18.18	2	18.18	
Malignant	9	81.81	9	81.81	
Defect side					
Right	4	36.36	3	27.27	
Left	7	63.63	8	72.72	
Defect class					
IIIc	2	18.18	1	9.09	
IIId	3	27.27	2	18.18	
IVc	2	18.18	3	27.27	
IVd	2	18.18	1	9.09	
IIb	1	9.09	1	9.09	
IVb	1	9.09	1	9.09	
IIIb	–	00	2	18.18	
Flap side					
Right	5	45.45	7	63.63	
Left	6	54.54	4	36.36	
Defect length	Mean ± SD		Mean ± SD		P value
	7.92 ± 1.49		8.80 ± 1.34		0.161

### Primary (aesthetic) outcomes evaluation

#### Differential surface area

The imaging analysis showed that the patients in the study group undergoing reconstructive surgery with CAD/CAM cutting guide showed improved aesthetic outcome (contour symmetry) in sagittal differential area with a mean of (241.39 ± 113.74 mm^2^), compared to patients in control group who were undergoing reconstructive surgery using CAD/CAM model with a mean of (358.70 ± 143.99 mm^2^). There was a significant difference between the two groups (P = 0.047) (Table 2).

**Table 2:** Comparison between study and control groups for sagittal and axial differential area in millimeter

Outcome	Study	Control	*P-*value
	Mean (in mm) ± SD	Mean (in mm) ± SD	
Sagittal surface area	241.39 ± 113.74	358.70 ± 143.99	0.047*
Axial surface area	163.44 ± 53.73	188.63 ± 59.21	0.300

Also, there was a slight improvement in the aesthetic outcome (in axial differential area) in the study group, with a mean of (163.44 ± 53.73 mm^2^), than in the control group with a mean of (188.63 ± 59.21 mm^2^). However, no significant difference was seen (P = 0.300) (Table 2).

#### Visual analogue scale

Subjective satisfaction with aesthetic results was higher in the study group, with a mean of (8.04 ± 0.68), than in the control group with a mean of (7.54 ± 0.85). There was no statistically significant difference between the two groups (P = 0.14) (Table 3).

**Table 3:** Comparison between study and control groups for Visual analogue scale and patient’s satisfaction score

Outcome	Study	Control	*P-*value
	Mean (score 1–10) ± SD	Mean (score 1–10) ± SD	
Visual analogue scale	8.04 ± 0.68	7.54 ± 0.85	0.14
Patient’s Satisfaction Score	8.09 ± 0.73	7.31 ± 0.92	0.039*

#### Patients’ satisfaction score

The postoperative aesthetic appearance evaluated by the patients was better in study group with the mean of (8.09 ± 0.73) than in the control group with the mean of (7.31 ± 0.92). There was statistically significant difference (P = 0.039) reflecting more aesthetic improvement in study group compared to control group (Table 3).

### Secondary outcomes (operation and ischemic time)

In the study group, the total operation time for the primary tumor surgery and reconstruction ranged from 636 to 702 min (mean = 678.81 ± 53.61 min), while in the control group, the total operation time ranged from 705.6 to 780 min (mean 777.18 ± 56.56 min). There was statistically significant difference between the two groups (P = 0.0004) (Table 4).

**Table 4:** Comparison between study and control groups for operation time and ischemic time (in min)

Outcome	Study	Control	*P-*value
	Mean (in min) ± SD	Mean (in min) ± SD	
Operation time	678.81 ± 53.61	777.18 ± 56.56	0.0004*
Ischemic time	133.18 ± 19.31	195.72 ± 20.67	0.000*

The mean ischemia time was (133.18 ± 19.31) min in the study group, while in the control group, the mean ischemic time was (195.72 ± 20.67) min. There was statistically significant difference between the two groups (P = 0.000) (Table 4). The ischemia time and the operation time were shorter in the study group than that in the control group.

### Surgical complications

The duration of the hospital stay ranged from 10–14 days. No donor site complications were observed; there was only mild restriction of shoulder movement on the harvested side, which completely resolved during the hospital stay. Otherwise, there was no significant postoperative morbidity at the donor site. There were no complications with the trans-axillary wound and the stitches were removed on postoperative day 7. There were no acute recipient site complications except one case of seroma formation, a minor infection of one flap and one patient was developed an oronasal fistula and underwent revision surgery to close the fistula by primary closure. None of the patients complained of significant pain or discomfort after a few months from the operation. In all cases, the transplanted bone had good healing and no medical complications occurred.

## Discussion

The maxilla is considered the most important skeletal bone that supports the midface; not only does the maxillary bone provide function, but it is also responsible for maintaining favorable aesthetics. Hence, maxillary defects following tumor ablation are usually complex as they affect soft tissue, bone and dentition. These deformities can have a significant effect on function, quality of life as well as the psychological wellbeing of patients due to the resultant atypical appearance and aesthetic derangement [8, 9].

As evident in the available literature, there have been various methods used for reconstruction of maxillectomy defects. Studies have described the use of obturator prosthesis with or without implant, local flaps (palatal, modified cheek), pedicled flaps (sternomastoid platysma cutaneous, deltopectoral, temporalis, vascularized cranial bone), and free flap [10]. Nevertheless, the use of these flaps to perform osteotomies, for restoring the natural appearances of the whole three-dimensional structures of the palatal and maxillary bones, poses a challenge. For this reason, the reconstructive surgeons are exploring the use of scapular free flaps for maxillary reconstruction [11].

The two factors that make the scapula the perfect bone for midrace reconstruction, specifically for reconstruction of the anterior maxillary face and alveolar ridge, are its shape and natural contour. The scapula can also be used for recreating the orbital rim and nasal aperture. Depending on how the scapula tip is oriented, it can mimic different parts of the midfacial skeleton to a great extent. For instance, when the scapular tip is horizontally placed, it can morphologically resemble the palate or orbital floor. On the other hand, if the scapular tip is positioned vertically, it can replace the anterior maxillary face. Hence, the scapula can be used for the reconstruction of the hard palate of the surface of the anterior maxillary wall [12].

Since the bone is formed of different components connected together, each component can be separately positioned, which can aid in repairing class III defects. Furthermore, harvesting of a skin paddle is also possible, when required. This can be used to fill intraoral defects and also used in cases of midfacial or paranasal soft-tissue defects. Other characteristics of the scapular tip are its long pedicle which can measure up to 20 cm and its triangular or round shape. These features allow the scapular tip to be used in reconstruction of various defects [13, 14]. Our choice is therefore justified by the above advantages of free scapular flap to be used for maxillary reconstruction.

In the case of conventional methods of maxillary reconstruction, the use of free hand for shaping the bone graft during surgery is considered difficult and time consuming. Regardless of the skills of the surgeons, the surgical outcomes of reconstruction can never be predicted [15].

To the best of our knowledge, only few studies have previously described the use of multiple computer-assisted techniques in oral and maxillofacial reconstruction, specifically in the reconstruction of extensive maxillary defects with free scapular flap. Therefore, this study reports one of the first series that use VSP in scapular free flap reconstruction in the maxillary region. Moreover, it is considered the only series that compares patients with and without VSP, particularly surgical cutting guide, in this setting.

Even though VSP has been described for maxillary reconstruction, this technique is still considered new, and not widely discussed in the present literature. This may be due to multiple reasons that make pre-operative virtual planning particularly difficult in this setting [16].

According to the published literature, up to date, there is no clinical trial that compared scapular free flap with CAD/CAM cutting guide versus scapular free flap without cutting guide in reconstruction of maxillary defect. In our study, we aimed to compare aesthetic outcomes and operation time of free scapular flap with and without CAD/CAM customized osteotomy guide for reconstruction of maxillary defects.

This study was conducted to improve aesthetics as well as other outcomes. For patients undergoing reconstruction of maxillary defects using free scapular flap, we believed that CAD/CAM technology using a customized cutting guide would be more successful in achieving these objects compared to using the same approach without a customized cutting guide.

In this study, 22 patients with maxillary tumors presented to the surgery department for the surgical resection and reconstruction. Of these 22 patients, 11 underwent reconstruction with free scapular flap using CAD/CAM surgical cutting guide (study group) and 11 underwent reconstruction with free scapular flap without surgical cutting guide (control group).

Regarding aesthetic outcome, which was evaluated objectively by digital computerized analysis in the form of contour symmetry and facial appearance for sagittal surface area, there was a statistically significant difference between the two groups (P < 0.05), with a more aesthetic improvement in the study group than that in the control group. Also, there was an improvement in aesthetic outcome in axial surface area in the study group than that in the control group, but without a significant difference (P > 0.05).

This improvement in aesthetic outcome was attributed to the use of CAD/CAM cutting guide in the study group. This cutting guide allowed the cutting saw to divide the bone with the desired shape, and to allow maximum points of bony contacts. As a result, proper bone adaptation and accurate anatomic position were achieved, which resulted in reduced differential surface area with enhancement of aesthetic outcomes.

It has become apparent how virtual planning has a favorable effect on maxillofacial reconstruction with regards to accurate placement of the graft and plate as well as providing better bone-to-bone contact. Comparing the preoperative plan to the post-operative clinical outcome revealed that the surgical cutting guides transferred a high level of precision to the execution of mandibular/maxillary and flap osteotomies [17–19].

The results of our study were in agreement with the results of three other studies [7, 20, 21] as they evaluated aesthetic outcome in patients with maxillary defects reconstructed by free scapula flap with CAD/CAM cutting guide and showed more successful aesthetic and functional outcomes, and a more satisfied patient.

Our results were similar to the results obtained by other studies [12, 15, 22–25]. They found that the use of VSP, particularly surgical cutting guide and precontoured plates, eased osteocutaneous flap molding and inset to better match the contours of the facial skeleton. This allowed for a more complex procedure to be successful, improved the restoration of the midface subunits and resulted in a more accurate approximation with normal bone anatomy and symmetry.

Contrary to the above statements, Chang et al. [26] believed that excellent accuracy could be achieved solely by a well-experienced microsurgeon, without VSP.

Aesthetic outcome was also evaluated subjectively by the patients and two evaluators (the surgeon and maxillofacial professional) using visual analogue scale and Patient’s Satisfaction Score. The patients were more satisfied with postoperative aesthetic appearance in the study group than that in the control group and there were statistically significant differences (P ˂ 0.05) reflecting more aesthetic improvement.

The results of the current study were in agreement with the results of [10, 20, 21, 27]. They revised planning protocol for reconstruction of the neomaxillary alveolar arch using either scapula or fibula free flaps, with aid of CAD/CAM cutting guide that was able to achieve a shape and contour that is consistent with a normal maxillary alveolar arch. All patients were satisfied with their postoperative appearance. In 2014, Rodby et al. [28] performed a systematic literature review and concluded that the increased accuracy resulting from use of VSP technology could result in possible increased patient satisfaction, which is in accordance with the results reported in our study.

In the study done by Bouchet et al., 2018, an interesting finding was that patients treated by the conventional approach were more satisfied with aesthetic and social activity outcome than patients treated by CAD/CAM approach. This finding contrasts with the present study [29].

Although subjective satisfaction with aesthetic results was slightly higher in the study group with the mean of (8.04 ± 0.68) than the control group with the mean of (7.54 ± 0.85). The aesthetic assessment by the surgeon and maxillofacial professional showed that there were no statistically significant differences between the two interventions groups (P = 0.14).

The total operation time in maxillary reconstruction was compared between (study group) utilizing scapular free flap with CAD/CAM osteotomy cutting guide and (control group) utilizing scapular free flap without osteotomy cutting guide. The tumor resection and the scapula osteotomy and shaping takes less time with the CAD/CAM osteotomy cutting guide technique. A shorter operative time is certainly more beneficial for the patient.

Our results show significant differences between the two groups with the ischemia time (P = 0.000*) and total operative time (P = 0.0004). The operative time and ischemia time were shorter in the study group than that in the control group. The decrease in total operation time was possibly a result of using the surgical guide to which the position and design of the osteotomies were transferred. The benefits of using this surgical guide were that it led the surgeon during surgery, which resulted in decreasing the operating room time, operator fatigue as well as the ischemia time.

The present study is in agreement with Culié et al., 2016 since both studies argued that the reduction in ischemia time may be attributed to the use of cutting guides and preformed plates which resulted in easier and faster surgery, and to the possibility of completing conformation with the flap still perfused at the donor site. On the other hand, without CAD/CAM cutting guide, conformation is usually completed at the cervicofacial receiver site before vascular anastomosis [30].

The results of the present study were in accordance with other reports by Wang et al. [6], Mazzola et al. [7] and Boukovalas et al. [20]. These authors found significant differences in operation and ischemic times when comparing the VSP cutting guide with the non-VSP group during reconstruction of maxillary and mandibular defects.

We agree with Kass et al., 2018 in that one of the main advantages of VSP planning is determining the actual position of the highest quality bone stock before surgery during the planning session. Moreover, VSP planning facilitates the inset of the flap by preventing the harvesting of a flap with excess osseous and soft tissue bulk, which limits the required intraoperative flap adjustments, minimizing the operation time [31]. In 2020, a study by Swendseid et al. showed that the difference between the mean operative times of the VSP and non-VSP groups (12.3 h vs 12.6 h, P = 0.70) was not significant, although it was slightly shorter with the VAS technique, which is in contrast with our result [23].

We agree with Sweeny et al., 2021 who discussed the limitations of VSP such as the increased number of preoperative steps and manipulations required, expensive outsourcing as well as added costs for healthcare. In addition, planning sessions are usually time-consuming, which may not align with the surgeons’ busy schedules. It would be understandable if the high cost of VSP was balanced out by less lengthy operative times and complications; however, this is not the case [12].

Furthermore, we agree with Pietruski et al., 2015 who stated that using cutting guides in certain situations can be useless; this is because sometimes the surgical plans, specifically the resection margins, can be altered intraoperatively to achieve total resection of tumors. Furthermore, cutting guides have to be manually positioned in a specific location and should ideally adhere to bone. This can act as a limitation in the case of large cutting templates, as massive dissection of the adjacent soft tissues would then be needed [32]. Mazzola et al. [7] believed that the pre-operative time dedicated to VSP has substantial advantages in terms of avoiding errors in flap configuration and reducing ischemia time after pedicle section.

Fortunately, using osteotomy guides ensures that estimations are no longer needed when attempting to shape the scapula into the neomaxilla as the accurate preoperative planning procedure enables highly precise alignment of bone. Combining patient specific planning before surgery with the use of pre-bent plating system means that the same drill holes which were used to mount the osteotomy guide to the scapula can be utilized to secure the scapula to the miniplate. For these reasons, CT-guided preoperative planning provides results that are specific to each individual patient and each clinical scenario encountered by the surgeon.

This study revealed how using CAD/CAM guide technology prevents the trial and error phase when attempting to shape the scapular bone flap to match the contour of the maxilla; thus, improving the surgical efficiency and reducing the time the patient has to be anesthetized within. As the surgeons become more experienced with this technology, the results will even become more favorable as the ischemic and operative times are predicted to be reduced as well as better aesthetic outcomes in complex cases of maxillary reconstructions.

## Conclusion

Necessity of using CAD/CAM prefabricated cutting guides facilitates scapular flap molding, placement and achieve precise maxillary reconstruction with a vascularized scapular flap after tumor ablation, as well as better aesthetic outcomes and decreased ischemic time and operating time.

## Data Availability

The datasets used and/or analyzed during the current study are available from the corresponding author on reasonable request.

## Abbreviations

CAD/CAM: Computer-aided design and computer-aided manufacturing
VSP: Virtual surgical planning
CT: Computed tomography
DICOM: Digital Imaging and Communications in Medicine
3D: 3-Dimensional
STL: Stereolithography
CDIA: Computerized Digital Imaging Analysis
VAS: Visual analogue scale
PSS: Patient’s Satisfaction Score
